# Gene expression profiling of pre-eclamptic placentae by RNA sequencing

**DOI:** 10.1038/srep14107

**Published:** 2015-09-21

**Authors:** Tea Kaartokallio, Alejandra Cervera, Anjuska Kyllönen, Krista Laivuori, The FINNPEC Core Investigator Group, Hannele Laivuori, Seppo Heinonen, Eero Kajantie, Juha Kere, Katja Kivinen, Anneli Pouta

**Affiliations:** 1 Medical and Clinical Genetics, University of Helsinki and Helsinki University Hospital, Helsinki, FI-00014, Finland; 2 Research Programs Unit, Genome-Scale Biology and Institute of Biomedicine, University of Helsinki, Helsinki, FI-00014, Finland; 3 Molecular Neurology Research Program, University of Helsinki, Helsinki, FI-00014, Finland; 4 Folkhälsan Institute of Genetics, Helsinki, FI-000290, Finland; 5 Department of Biosciences and Nutrition, Center for Innovative Medicine, and Science for Life Laboratory, Karolinska Institutet, Stockholm, Sweden; 6 Obstetrics and Gynaecology, University of Helsinki and Helsinki University Hospital, Helsinki, FI-00014, Finland; 7 Institute for Molecular Medicine Finland, University of Helsinki, Helsinki, FI-00014, Finland; 8 Department of Obstetrics and Gynecology, Kuopio University Hospital, and University of Eastern Finland, Kuopio, Finland; 9 Department of Chronic Disease Prevention, Diabetes Prevention Unit, National Institute for Health and Welfare, Helsinki, Finland; 10 Children’s Hospital, Helsinki University Hospital and University of Helsinki, Helsinki, Finland; 11 Department of Obstetrics and Gynaecology, MRC Oulu, Oulu University Hospital and University of Oulu, Oulu, Finland; 12 Wellcome Trust Sanger Institute, Wellcome Trust Genome Campus, Hinxton, Cambridge, UK; 13 Division of Cardiovascular Medicine, University of Cambridge, Cambridge, UK; 14 Department of Children, Young people and Families, National Institute for Health and Welfare, Oulu, Finland

## Abstract

Pre-eclampsia is a common and complex pregnancy disorder that often involves impaired placental development. In order to identify altered gene expression in pre-eclamptic placenta, we sequenced placental transcriptomes of nine pre-eclamptic and nine healthy pregnant women in pools of three. The differential gene expression was tested both by including all the pools in the analysis and by excluding some of the pools based on phenotypic characteristics. From these analyses, we identified altogether 53 differently expressed genes, a subset of which was validated by qPCR in 20 cases and 19 controls. Furthermore, we conducted pathway and functional analyses which revealed disturbed vascular function and immunological balance in pre-eclamptic placenta. Some of the genes identified in our study have been reported by numerous microarray studies (*BHLHE40*, *FSTL3*, *HK2*, *HTRA4*, *LEP*, *PVRL4*, *SASH1*, *SIGLEC6*), but many have been implicated in only few studies or have not previously been linked to pre-eclampsia (*ARMS2*, *BTNL9, CCSAP*, *DIO2*, *FER1L4*, *HPSE*, *LOC100129345*, *LYN*, *MYO7B*, *NCMAP*, *NDRG1*, *NRIP1, PLIN2*, *SBSPON, SERPINB9, SH3BP5*, *TET3*, *TPBG*, *ZNF175*). Several of the molecules produced by these genes may have a role in the pathogenesis of pre-eclampsia, and some could qualify as biomarkers for prediction or detection of this pregnancy complication.

Pre-eclampsia, a vascular pregnancy disorder characterised by new-onset hypertension and proteinuria, affects approximately 3–5% of all pregnancies^1^,^2^. The disease causes over 63 000 maternal deaths yearly, and is one of the major causes for premature birth^3^. Pre-eclampsia is considered to have a multifactorial, polygenic etiology that, despite intensive research, remains elusive. The syndrome likely includes several subtypes^4^,^5^. According to a prevalent theory, early-onset pre-eclampsia accompanied with fetal growth restriction involves poor placental development, whereas in the late-onset form pre-existing maternal cardiometabolic risk factors might play a more significant role^6^.

For certain subtypes of pre-eclampsia, there is strong evidence for the involvement of disturbed placental development^7^,^8^. During normal placentation, fetal extravillous trophoblast cells invade into the uterine wall, and together with other modifying cells transform maternal spiral arteries wider and less resistant, and thus better adapted for supplying adequate blood flow for the fetus. In pre-eclampsia the extravillous trophoblast invasion is shallower and the modification of arteries insufficient^7^,^9^, which leaves the arteries narrower and more contractile, and subsequently increases resistance and fluctuations in placental blood flow^10^,^11^,^12^. Due to still unknown mechanisms that may involve cell necrosis caused by oxidative and mechanical shear stress, pre-eclamptic placenta releases excess amount of placental material into maternal circulation^13^,^14^. Placental factors, such as antiangiogenic soluble fms-like tyrosine kinase-1 (sFlt-1)^15^,^16^, are believed to trigger systemic inflammation and endothelial dysfunction that manifest as the maternal symptoms of hypertension and proteinuria^17^,^18^.

As placenta is centrally involved in the pathophysiology of pre-eclampsia, gene expression in pre-eclamptic placentae has been studied extensively with microarrays^19^,^20^,^21^. According to a recent systematic review, the genes most frequently differently expressed, *LEP* and *FLT1*, had been found in only third of the studies^19^, indicating inconsistency between the expression studies reviewed. This might be partly explained by the phenotypic and etiologic heterogeneity of pre-eclampsia, as well as by the differences in study design for factors that affect placental gene expression such as gestational weeks, sex of the child or method of delivery. Moreover, technical reasons including differences in placental sampling site, sample handling, microarray platforms and statistical methods add further heterogeneity between the studies.

To the authors’ knowledge, this is the first study to apply RNA sequencing (RNA-seq) in studying gene expression in pre-eclamptic placenta. Studies comparing performance of microarrays to that of RNA-seq have revealed several advantages of the latter^22^,^23^,^24^,^25^. Among these is the wider dynamic range of RNA-seq, due to which it is more sensitive in detecting genes with low expression level. Moreover, RNA-seq is not probe-based and therefore has a better genomic coverage. Thus, RNA-seq outperforms microarrays in sensitivity and is able to survey larger amount of genes, giving this method potential to reveal differential expression for genes not previously linked with pre-eclampsia. Identifying genes with altered expression in pre-eclamptic placenta would help to discover molecular mechanisms involved in the development of this common pregnancy disorder, as well as molecules qualifying for the prediction and detection of this disease.

## Results

### Clinical characteristics

Clinical characteristics of the study subjects are presented in Table 1. In addition to diagnostic criteria of pre-eclampsia, the cases and controls in the RNA-seq differ statistically significantly for parity, gestational age at birth, relative birth weight and delay from detachment of placenta to sampling. In the sample set used for the quantitative PCR (qPCR) validation, the case and control groups differ for parity, relative birth weight and delay from detachment of placenta to sampling (Table 1).

### Quality control of the RNA-seq data

On average, 1.85% of the original RNA-seq reads were filtered out in the quality filtering step, the percentage of filtered reads per pool being between 1.6 and 2.0%. After trimming and filtering, the quality of each forward and reverse pool was assessed. Read lengths for the forward data were between 25 and 101 bases for every pool whereas for the reverse data read length ranged between 25–90 and 25–101 bases depending on the pool. The average GC content per pool was 48–50%. The number of reads per pool after trimming and filtering ranged from 29 358 515 to 39 060 015 reads, the average of all pools being 32 670 939. The pools contained 64.6–73.6% duplicate reads, the average of all pools being 68.8%. The quality metrics for the RNA-seq data are shown in Supplementary Table S1.

After quality trimming and filtering, the reads were aligned to the reference genome. Quality metrics for the alignment are shown in Supplementary Table S2. Approximately 94% of the reads mapped with mate. For only 1% of these reads, the paired reads mapped to different chromosomes, indicating that majority of the reads aligned correctly. On average, 91% of the alignments were unique, aligning to only one position in the reference genome. Of the reads mapping to the annotated genes, on average 14.7% mapped to the top 10 most highly expressed genes and 19.8% to the top 20 most highly expressed genes. These numbers may partly explain the high numbers of duplicate reads as the abundant transcripts are more likely to be sequenced multiple times. Hence, many of the duplicate reads probably stem from high gene expression rather than from PCR duplication, therefore representing a true biological phenomenon.

### Differential gene expression in the RNA-seq

**Figure 1:**
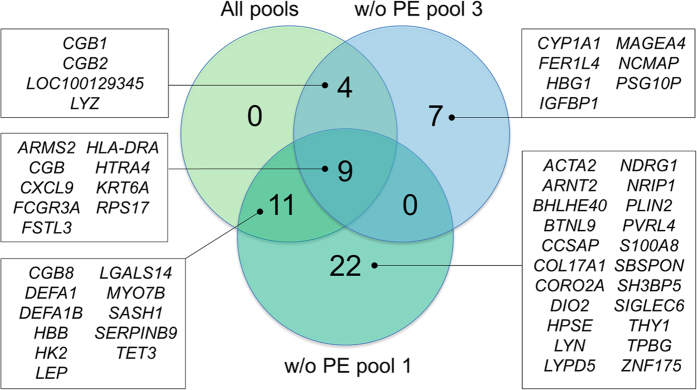
Differentially expressed genes in the RNA-seq by subanalysis. Placental samples of nine pre-eclamptic and nine non-pre-eclamptic women were analysed in pools of three. Three approaches were used in the analysis: 1) All samples were included in the analysis, 2) Pre-eclampsia pool 1 was excluded from the analysis and 3) Pre-eclampsia pool 3 was excluded from the analysis. PE = pre-eclampsia.

**Figure 2:**
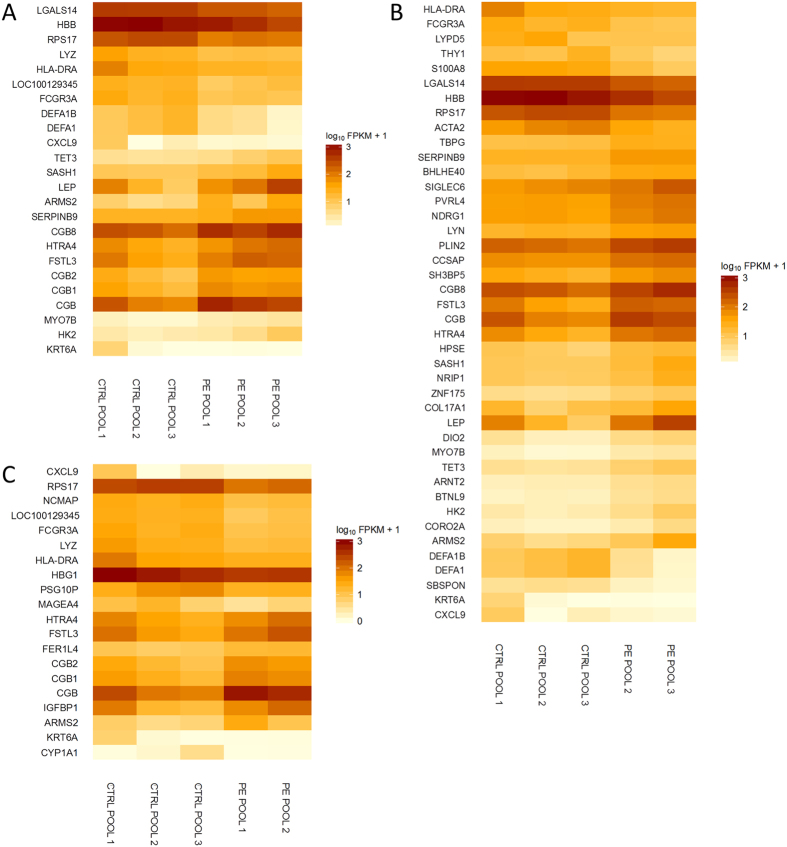
Heat maps by subanalysis showing the log_10_FPKM + 1 values of 53 transcripts differentially expressed in the RNA-seq. Placental samples of nine pre-eclamptic and nine non-pre-eclamptic women were analysed in pools of three. CTRL = control, PE = pre-eclampsia. FPKM = Fragments Per Kilobase of exon per Million fragments mapped. (**A**) All samples included in the analysis (**B**) Pre-eclampsia pool 1 excluded from the analysis (**C**) Pre-eclampsia pool 3 excluded from the analysis.

The differential gene expression was tested utilising three strategies. First, all the pre-eclampsia pools were compared against all the control pools. Second, the pre-eclampsia pools 2 and 3 were compared against the control pools. The pre-eclampsia pool 1 was excluded as it contained a Trisomy 21 placenta (information on Trisomy 21 diagnoses were not available at the time of the sample selection), and also because it may represent less severe pre-eclampsia phenotype as all the placental samples in this pool originate from term deliveries without fetal growth restriction. Third, the pre-eclampsia pools 1 and 2 were compared against the control pools. The pre-eclampsia pool 3 was excluded because it differed most from the control pools for gestational age. From these three subanalyses, we obtained altogether 53 genes that were statistically significantly differently expressed between the case and control groups, when the Y chromosomal genes were excluded, as the fetal sex distribution between the groups was not equal. The overlap in the results from the different subanalyses is illustrated in Fig. 1. The differently expressed genes along with their summarised function, protein localisation, and expression difference (log_2_ fold change) are listed in Table 2, and the expression profiles of the genes per subanalysis are shown in Fig. 2. The detailed result files containing differently expressed genes in each subanalysis are found in Supplementary Table S3.

Altogether 577 distinct genes had a log_2_ fold change ≥1 or ≤ −1 (two-fold difference) in at least one of the subanalyses, when the Y chromosomal genes were again excluded. Among these genes are *FLT1*, *INHBA*, *CRH, PAPPA2* and *LHB*, genes often differently expressed in pre-eclampsia. The overlap in the genes with a log_2_ fold change ≥1 or ≤ −1 between the subanalyses is illustrated in the Supplementary Fig. S1.

### Pathway and functional analyses

#### Gene set enrichment analysis

Gene set enrichment analysis (GSEA) was conducted for each subanalysis independently. The analysis was carried out twice using two separate gene sets (hallmark gene sets and canonical pathways gene sets). With the hallmark gene sets 16, 10 and 10 pathways, and with the canonical pathways gene sets 72, 60 and 84 pathways were statistically significant in the analyses with all the pools included, with pre-eclampsia pool 1 excluded and with pre-eclampsia pool 3 excluded, respectively. Immunological as well as autoimmune and inflammatory disease pathways were enriched among significant pathways. Also signal transduction pathways that control growth and differentiation were abundant. Other significant pathways were related to developmental processes such as extracellular matrix and tissue remodelling, angiogenesis and epithelial-mesenchymal transition as well as to DNA damage response and reactive oxygen species. The significant pathways from the analyses utilising hallmark gene sets are shown in Table 3 and the pathways from the analyses with canonical pathways gene sets in Supplementary Table S4.

### Ingenuity canonical pathway analysis

The genes with at least two-fold increase or decrease in expression in the pre-eclamptic placentae compared to the control placentae (log_2_ fold change ≥1 or ≤ −1) were included in the Ingenuity Pathway analysis (IPA) conducted separately for each subanalysis. Altogether 44, 60 and 41 canonical pathways were statistically significant in the analysis with all the pools included, with pre-eclampsia pool 1 excluded and with pre-eclampsia pool 3 excluded, respectively. Multiple immunological functions from both innate and adaptive immune systems were altered in pre-eclamptic placenta. Many of the significant pathways were related to autoimmune disease, such as rheumatoid arthritis and systemic lupus erythematosus. Also pathways related to migration, tissue development and angiogenesis as well as to stress response and signalling were enriched. The complete list of the pathway analysis results can be found in Supplementary Table S5.

#### Ingenuity pathway functional analysis

The disease and functional annotations that were most significant in all the subanalysis are related to vast amount of immunological functions, tissue development, cell movement and cell communication and signalling. Other significant annotation categories include autoimmune and inflammatory diseases, cell death and cell proliferation, oxidative stress, cardiovascular disease and cardiovascular system development and function. The categories that included most disease and function annotations in the functional analysis and selected additional categories are presented in Supplementary Table S6 and the complete lists of the significant annotations can be found in Supplementary Table S7.

#### qPCR validation

**Figure 3:**
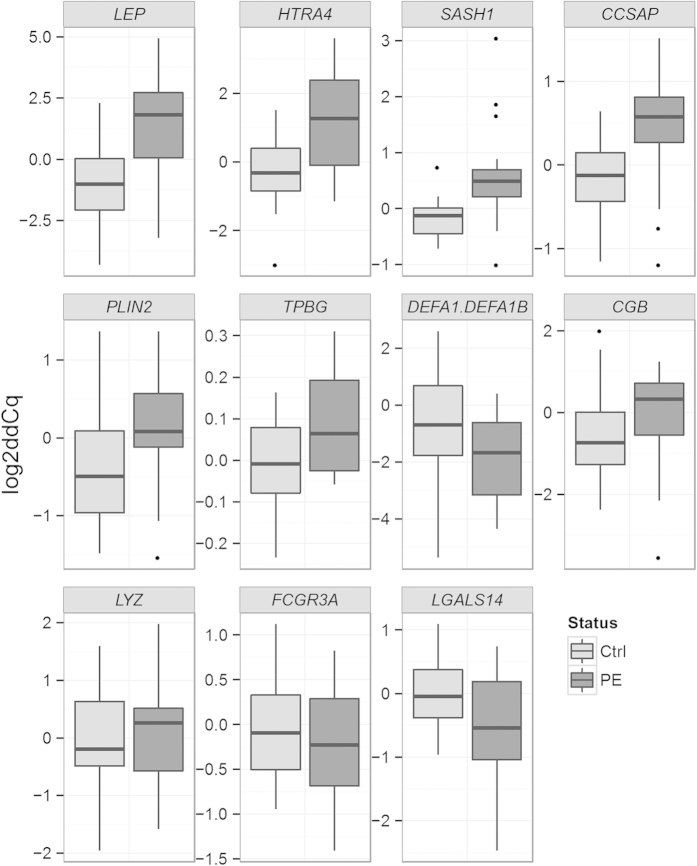
Comparison of the placental gene expression in the qPCR validation between 20 pre-eclamptic and 19 non-pre-eclamptic women. Eight pre-eclamptic and nine non-pre-eclamptic women from the RNA–seq were included in the validation. The genes were selected for validation based on differential expression in the RNA-seq. ddCq = delta delta Cq, Cq value normalised for geometric mean of reference genes and a calibrator.

**Figure 4:**
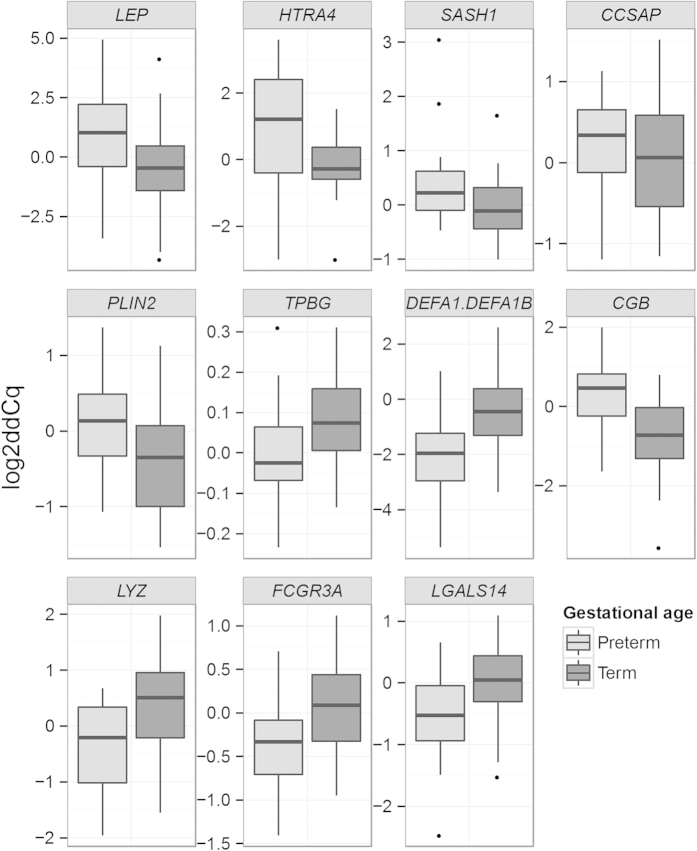
Comparison of the gene expression in the qPCR validation between preterm (11 from pre-eclamptic pregnancies and 8 from pregnancies without pre-eclampsia) and term (9 from pre-eclamptic pregnancies and 11 from pregnancies without pre-eclampsia) placentae. ddCq = delta delta Cq, Cq value normalised for geometric mean of reference genes and a calibrator.

Of the total number of 53 genes that were differently expressed in the RNA-seq, 12 were originally selected for further qPCR validation. Due to the sequence similarity between *DEFA1* and *DEFA1B*, the Taqman assays are not able to separate their expression from one another and the same assay detects expression of both making the number of assays utilised in the qPCR validation 11. In the qPCR analysis we focused on distinguishing the disease-related gene expression differences from the differences caused by variability in gestational age. The effect of pre-eclampsia and gestational age on gene expression was analysed using both non-parametric permutation test and two-way ANOVA. The continuous variable gestational age was recoded into a dummy variable (preterm/term). In the validation, five of the 11 genes (*CCSAP, HTRA4*, *LEP, PLIN2, SASH1*) were differently expressed between the cases and controls. For five genes (*CGB, DEFA1/DEFA1B1, FCGR3A, LGALS14, LYZ*) the expression difference was dependent on gestational weeks. The expression level of *TPBG* was affected both by gestational weeks and pre-eclampsia. For *HTRA4*, the expression level in the pre-eclamptic group was affected by weeks of gestation, whereas for the control group this effect was not seen. This suggests that the increased *HTRA4* expression might be related to the pre-eclampsia phenotype associated with preterm delivery and fetal growth restriction. Indeed, for *CCSAP*, *HTRA4*, *LEP*, *PLIN2* and *SASH1* log_2_Cq values inversely correlate with birth weight normalised for weeks of gestation in the pre-eclamptic group (data not shown), indicating that the expression level of these genes might be related to the amount of placental pathology involved. The results from the qPCR validation are shown in Table 4 and in Figs 3 and 4. Excluding outliers from the analysis did not change the result significantly. Excluding diabetic patients from the analysis did not cause significant changes for majority of the genes. The most notable difference in this analysis was that the expression of *PLIN2* did not differ statistically significantly between pre-eclamptics and controls, but differed between preterm and term placenta (Supplementary Table S8).

## Discussion

In the present study, we conducted an RNA-seq analysis to compare gene expression between pre-eclamptic and healthy placenta, and found 53 genes that differed in expression between the groups. The genes include those previously identified by numerous microarray studies (e.g. *BHLHE40*, *FSTL3*, *HK2*, *HTRA4*, *LEP*, *PVRL4*, *SASH1*, *SIGLEC6*), but also those that have been reported by only few studies or have not previously been linked to pre-eclampsia (*ARMS2*, *BTNL9, CCSAP*, *DIO2*, *FER1L4*, *HPSE*, *LOC100129345*, *LYN*, *MYO7B*, *NCMAP*, *NDRG1*, *NRIP1, PLIN2*, *SBSPON, SERPINB9, SH3BP5*, *TET3*, *TPBG*, *ZNF175*).

The results from expression array studies on pre-eclamptic placentae have recently been summarised in a systematic review^19^, in a meta-analysis^20^, and in a large-scale aggregate analysis^5^. Kleinrouweler and others^19^ reviewed 30 studies that compared placental gene expression between pre-eclamptic and non-pre-eclamptic pregnancies, and defined a meta-signature of 40 transcripts that were reported to be differentially expressed in pre-eclampsia by at least three studies. Of the 53 genes identified in our study, nine (17.0%) (*BHLHE40*, *CGB*, *FSTL3*, *HTRA4*, *IGFBP1, LEP*, *PVRL4*, *SASH1* and *SIGLEC6*) belong to this meta-signature. Furthermore, of the 577 genes that had a log_2_ fold change ≥1 or ≤ −1 in at least one of our subanalyses, 15 (2.6%) belong to the meta-signature. These genes with noticeable although not necessarily statistically significant expression difference between the pre-eclamptic and non-pre-eclamptic placentae include many that have been implicated in previous studies, such as *FLT1*, *INHBA*, *CRH, PAPPA2* and *LHB*. Important limitation in the review by Kleinrouweler and others is that only some of the studies list a complete set of the differently expressed genes while many report just selected genes, which may bias the results. Vaiman and others^20^ compared mRNA levels between pre-eclamptic and control placentae by utilising raw data from six publicly available microarray datasets, and identified 67 genes that were upregulated and 31 genes that were downregulated in pre-eclamptic placentae in at least four out of the six datasets. Fourteen (26.4%) of our differently expressed genes (*BHLHE40*, *CCSAP*, *FSTL3*, *HK2*, *HTRA4*, *LEP, LYN*, *NDRG1*, *NRIP1*, *PVRL4*, *SASH1*, *SH3BP5*, *SIGLEC6* and *TPBG*) and 21 (3.6%) of the genes with two-fold expression difference in our data overlapped with these 98 genes. Leavey and others^5^ aggregated seven microarray data sets to generate a large set of 173 samples including 77 pre-eclamptics. They were able to separate three distinct subclasses of pre-eclampsia based on gene expression, and identified 1295 genes that showed altered expression in pre-eclamptic placentae compared to controls, as well as 1329 genes whose expression was altered in at least one of the three subclasses they found. Twenty-six (49.1%) of the genes differently expressed in our study were differently expressed in at least one of their analysis (*ACTA2*, *ARNT2*, *BHLHE40*, *BTNL9*, *CCSAP*, *CGB1*, *COL17A1*, *CORO2A*, *DIO2*, *FSTL3*, *HK2*, *HTRA4*, *LEP*, *LYN*, *LYPD5*, *MYO7B*, *NDRG1*, *NRIP1*, *PLIN2*, *PVRL4*, *SASH1*, *SERPINB9*, *SH3BP5*, *SIGLEC6*, *TPBG*, *ZNF175*). Furthermore, of the genes with log_2_ fold change between −1 and 1 in our analysis, 104 (18.0%) were reported as differently expressed in their study. These comparisons show that our study is in high concordance with previous microarray studies, and provides a valuable validation of previous results with a novel method. In addition, our study highlights genes not commonly reported by previous studies, such as *FER1L4*, *NCMAP*, *RPS17*, *SBSPON*, and *TET3*.

The pathway and functional annotation analyses in our data show several biological processes that are disturbed in pre-eclamptic placenta. The enrichment of immunological pathways and annotations reflects disturbed immunological balance and inflammation in pre-eclamptic placenta. In line with this, annotation analysis highlights multiple autoimmune, inflammatory and immunological diseases, which suggests that they might share partly common disease mechanisms with pre-eclampsia. Immune system molecules with altered expression in our study also participate in a wide array of developmental processes in placenta such as the development of haematological and vascular systems. Our results clearly show unbalance in many tissue development and homeostasis related processes such as cell death, proliferation, angiogenesis, migration and signalling. Many of the significant annotations including occlusion of artery, atherogenesis, atherosclerosis, vascular lesion, hypertension and oxidative stress are related to cardiovascular disease, emphasising common mechanisms between pre-eclampsia and other vascular diseases. Taken together, our results underline the intertwined processes of impaired vascular system development and function and immunological disturbance as most characteristic features of pre-eclamptic placenta. These results are in line with the interpretation of pre-eclampsia pathology depicted in the review by Staff and others^26^.

The qPCR expression levels of *CCSAP*, *HTRA4*, *LEP*, *PLIN2* and *SASH1* inversely correlated with gestational age –normalised birth weight in the pre-eclamptic group, suggesting that the altered expression of these genes might be related to the pre-eclampsia phenotype involving impaired placental development. For *HTRA4*, a gene that encodes a serine peptidase responsible for degradation of misfolded proteins, there are previous findings in accordance with this interpretation. In addition to elevated HTRA4 mRNA and protein expression in pre-eclamptic placentae, serum HTRA4 levels are increased in pre-eclamptics, being higher in early-onset cases and inversely correlating with weight of the baby and placenta^27^. This provides support for the hypothesis according to which elevated *HTRA4* expression may be related to the early-onset pre-eclampsia associated with fetal growth restriction.

To the authors’ knowledge, this is the first study utilising RNA-seq to explore placental gene expression differences between pre-eclamptic and non-pre-eclamptic pregnancies. In the RNA-seq, we included only placentae from C-sections without contractions in order to exclude the effect of labour on gene expression. Placental samples utilised in the study were collected according to a well-defined protocol and pooled in groups of three. Pooling potentially minimizes the effect of subject-to-subject variation and facilitates the detection of gene expression differences present in majority of the pre-eclamptic placentae. On the other hand, in studies where pooled samples are untagged individual samples cannot be analysed separately, outliers cannot be identified and weaker signals present in only subset of the samples might be missed. Limitations of the study include the difference in gestational age between the cases and controls and a modest sample size in the RNA-seq, issues that were partly addressed by validating a subset of the differently expressed genes in a larger sample set with more comparable gestational age between the cases and controls, and also by studying the dependence of the gene expression on gestational age in the validation. Difference in gestational age between cases and controls is a common issue in expression studies of pre-eclamptic placentae: women with severe pre-eclampsia tend to deliver prematurely, whereas healthy pregnant controls usually deliver at term. Including premature non-pre-eclamptic placentae as controls does not provide a non-problematic solution because prematurity might be caused by infection or other underlying condition that might severely affect placental gene expression. As we were not able to control for gestational age in the RNA-seq, some of the observed expression differences might be caused by this factor, as seen in the qPCR validation. One of the pre-eclampsia pools in the RNA-seq contained a Trisomy 21 placenta, which may increase the phenotypic heterogeneity of the pool. To circumvent this issue, we analysed the RNA-seq data also without this pool. The median of the delay from detachment of placenta to sampling differed 20 minutes between the cases and controls in the RNA-seq, but this is unlikely to be a major concern as RNA is shown to be stable for as long as 48 hours in intact placental tissue stored at +4^28^.

Data from differently expressed transcripts in pre-eclamptic placenta is accumulating, and could be exploited in clinical applications. Many of the molecules could potentially be deployed in diagnosing pre-eclampsia or as predictive biomarkers to detect women at risk of the disease. Furthermore, these markers could be used to recognise subphenotypes of pre-eclampsia. Secreted proteins are of special interest for these purposes, but also novel approaches such as sequencing of fetal RNA from maternal blood (recently reviewed by Oudejans^29^) provide intriguing options for development of screening tests. Genome-wide or targeted RNA-seq from maternal blood would also enable utilising large number of non-protein-coding transcripts (in our data e.g. *FER1L4* and *LOC100129345*). Although developing these kinds of tests will require solving of various technical problems and gathering of more detailed knowledge on placental gene expression in pre-eclampsia as well as knowledge on temporal gene expression during placental development, this approach could offer a large-scale screening method for predicting pre-eclampsia risk or clinical outcome.

In this first RNA-seq study on pre-eclamptic placentae we identified several genes involved in the biological processes relevant for the development of pre-eclampsia, such as immunological and vascular functions. The genes likely include those whose distorted expression is causative and directly involved in the development of pre-eclampsia as well as those whose expression levels change secondary to pathological condition seen in pre-eclamptic placenta. To separate causative expression differences from secondary changes, it would be ideal to study early-pregnancy placental samples collected before the clinical onset of the disease. These samples are due to obvious ethical reasons challenging to collect, but surplus from diagnostic samples obtained at the first trimester of pregnancy could provide a solution for future studies. Many of the proteins or transcripts produced by the genes with altered expression in this study could show potential as pre-eclampsia biomarkers, and the suitability of these molecules for predictive or diagnostic purposes should be evaluated in future studies.

## Materials and Methods

### Study subjects

The Finnish Genetics of Pre-eclampsia Consortium (FINNPEC) cohort^30^ includes placental samples from 102 pre-eclamptic and 79 non-pre-eclamptic pregnancies, of which 17 pre-eclamptics and 11 controls met our criteria for the RNA-seq. Nine pre-eclamptic patients and nine healthy pregnant women were selected for the RNA-seq, and the samples were pooled in pools of three. The samples were pooled in order to maximize the number of placental transcriptomes that could be sequenced with the resources available. All the cases in pre-eclampsia pool 3, and one case in the pre-eclampsia pools 1 and 2 had an early-onset pre-eclampsia (onset <34 + 0 weeks of pregnancy). The pre-eclampsia pools 1, 2 and 3 consist of placental samples from 38–39, 34–36 and 33 weeks of gestation, respectively, and the control pools from 38–39 weeks of gestation. Only term control placentae were utilised in the RNA-seq because in prematurely delivered placentae gene expression might be affected by infection or other unknown factors. Only women who delivered by elective or urgent C-section without labour were included to minimize the effect of delivery method on gene expression. Placental samples taken within 1.5 hours of placental detachment were considered eligible. Exclusion criteria applied were chronic hypertension, cardiovascular disease, diabetes, chronic inflammatory, autoimmune, haemolytic and renal diseases, hepatitis, placental ablation, chorioamnionitis, and multiple pregnancy. Additionally, placental insufficiency, small-for-gestational age baby (SGA) and gestational hypertension were considered as exclusion criteria for the controls. After the sequencing had been performed, the pre-eclampsia pool 1 was found to contain a Trisomy 21 placenta.

The sample set for the qPCR validation consists of placental samples from 20 pre-eclamptic patients and 19 non-pre-eclamptic women from the FINNPEC cohort. The validation sample set includes the original cases and controls from the RNA-seq, except for the Trisomy 21 placenta. The exclusion criteria were otherwise the same as in the RNA-seq, but diabetic women, women who had given vaginal birth and non-pre-eclamptic women with preterm delivery were included in the validation sample set to increase the sample size.

The diagnoses of pre-eclampsia were ascertained based on medical records with the following criteria: systolic blood pressure ≥140 mmHg and/or diastolic blood pressure ≥90 mmHg with new-onset proteinuria (≥0.3 g/24 hours) after 20 weeks of gestation^31^. Preterm delivery is defined as a delivery occurring before 37 weeks + 0 days of pregnancy. Birth weights below −2.0 SD units (birth weight relative to sex and length of gestation) according to Finnish standards^32^ are classified as SGA. Placental insufficiency is defined as uterine artery resistance index or pulsatility index > +2 SD units. The diagnoses were verified independently by a research nurse and a study physician.

### Ethical approval

All study participants have provided a written informed consent, and the study protocols have been approved by the Coordinating Ethics Committee of the Hospital District of Helsinki and Uusimaa. All experiments were performed in accordance with the approved guidelines.

### Sample collection and RNA extraction

The placental samples were collected according to a 9-site protocol where placenta is divided in nine sections, and a biopsy sample is harvested from each of these sections. The samples were obtained shortly after detachment of placenta: 70% of the samples were collected within half an hour, 92.5% within an hour and the remaining three samples within 80 minutes of placental detachment. The samples were subsequently stored in RNA later solution. For each study sample, RNA was extracted from two placental fragments (sites 1 and 8 for the RNA-seq and sites 2 and 4 for the qPCR validation) that were pooled together before the extraction. The RNA was extracted using MirVana miRNA Isolation Kit (Invitrogen) (for the RNA-seq) or PureLink® RNA Mini Kit (Invitrogen) (for the qPCR validation), and stored in −80 °C. Integrity of the RNA was evaluated with Bioanalyzer 2100 (Agilent).

### RNA sequencing

The placental transcriptomes were sequenced in Science for Life Laboratory (Stockholm, Sweden) with the Illumina sequencing platform. Before the sequencing, the RNA samples were pooled in pools of three. The RNA libraries were constructed with Illumina’s TruSeq RNA sample preparation kit. The clustering was performed on a cBot cluster generation system using a HiSeq paired-end read cluster generation kit according to the manufacturer’s instructions. The pools were sequenced on an Illumina HiSeq 2000 as paired-end reads to 100 bp. The sequencing runs were carried out according to the manufacturer’s instructions. Base conversion was performed using Illumina’s OLB v1.9.

### RNA-seq data analysis

The quality of the raw sequence reads was determined with the FastQC and PRINSEQ tools integrated in the Chipster software^33^. The reads were trimmed with Trimmomatic (v. 0.22)^34^. Bases with quality value <20 were cut off from the start and end of the reads and adapter sequences and reads shorter than 25 bp were removed. Reads containing >2 undetermined bases (N) were removed with the Chipster integrated PRINSEQ tool. After trimming and filtering, the reads were aligned to the reference genome (hg19) with Tophat (v. 2.0.4)^35^. The UCSC hg19 genome sequence indexes and the GTF transcript annotation files provided by Illumina were used in the alignment. A transcriptome index was built with the control pool 3 and the same index was subsequently utilised for the other alignments. The expected mean inner distance between mate pairs was estimated from the average total fragment size for each pool. 50 bp was set as the standard deviation for the distribution on inner distances between mate pairs. Otherwise, default settings were used for the alignment. The alignments were examined with SAMtools^36^ and HTSeq^37^ and visualised with the Chipster genome browser. Transcript assembly for each individual pool was conducted with Cufflinks (2.0.2)^38^. Multi read correct (-u) and upper quartile normalisation (-N) options were used in the assembly. The assemblies were merged together with Cuffmerge. The case and control groups were tested for differential gene expression with Cuffdiff. In the Cuffdiff run, multi read correct option (-u) was utilised and the tool was provided with the multifasta file the reads were mapped to (-b) to improve accuracy of transcript abundance estimates. In the differential expression analysis, a q value (an FDR corrected p value) of <0.05 was considered statistically significant. The results were visualised with CummeRbund^39^ in the R environment.

Pathway and functional annotation analyses were conducted separately for each subanalysis using GSEA^40^,^41^ and IPA (QIAGEN) software. The gene lists included in the GSEA analysis contained all the genes from the Cuffdiff differential gene expression result files except for the Y chromosomal ones and the ones for which Cuffdiff had not conducted differential expression test (test status FAIL or NOTEST). The analyses were performed with the GSEAPreranked tool, and the log_2_ fold change values were used as ranking values for the genes. Analyses were run twice using two different gene sets separately: the hallmark gene sets and the canonical pathways gene sets. The hallmark gene sets represent specific well-defined biological processes or states and have been created by computational methodology based on identifying gene set overlaps. Canonical pathways gene sets have been collected from pathway databases and have usually been gathered by domain experts. An FDR q value of <0.05 was considered significant in the GSEA analyses. For the analysis conducted with IPA, genes with a log_2_ fold change ≥1 or ≤ −1 were included. As for the GSEA analyses, the analyses were conducted separately for each subanalysis. Genes and endogenous chemicals were included as a reference set, and only experimentally observed relationships were considered. A p value of <0.05 or a corresponding –log(p value) of 1.3 were considered significant in the IPA analyses.

### Selection of the genes for the qPCR validation

The expression patterns of the genes that were differentially expressed between the cases and controls in the RNA-seq were visually evaluated using the Chipster genome browser and heat maps produced with CummeRbund. Based on this evaluation, we excluded genes that showed clearly overlapping expression pattern between the case and control pools. For the qPCR validation we selected genes that showed consistent differential expression across the case pools compared to the control pools, and preferred the genes with expression level large enough to be detected with qPCR. In addition, functional relevance of the genes for pre-eclampsia was considered in the selection process.

### Reverse transcription and qPCR

After the RNA extraction, TURBO DNA-free (Invitrogen) DNAase treatment was performed for each sample according to the manufacturer’s instructions to degrade any genomic DNA. A microgram of placental RNA was reverse transcribed to cDNA with High Capacity RNA-to cDNA Kit (Invitrogen) according to the manufacturer’s instructions. Duplicate reverse transcription reactions were prepared from each sample.

The qPCR validation was performed with Taqman chemistry (TaqMan® Fast Universal PCR Master Mix (2X), No AmpErase® UNG (Applied Biosystems)). The target genes and the Taqman assays utilised are listed in Supplementary Table S9. Reaction mixes were prepared according to the manufacturer’s instructions, and each sample was run in duplicate in the 7500 Fast Real-Time qPCR machine (Applied Biosystems) with the protocol for Taqman Fast chemistry. Before conducting the actual experiments, six point standard curves with 1:5 dilutions, except 1:3 dilution for *FCGR3A*, were prepared for each of the target genes to determine the linear range and the most suitable dilution (Supplementary Table S9). Endogenous control genes were selected among five candidates (*TOP1*, *PPIA*, *YWHAZ*, *UBC* and *TBP*) by studying the expression level of these genes in eight cases and in eight controls representative of the whole sample set, and evaluating stability of their expression by the geNorm^42^ and NormFinder^43^ software. *TBP* and *YWHAZ* were most stably expressed genes in our dataset, and were selected for endogenous controls. A cDNA pool constructed from the 19 control samples was utilised as a calibrator sample in the qPCR runs.

### qPCR data analysis

PCR efficiencies were determined with LinRegPCR^44^,^45^, and Cq values were corrected for efficiency and normalised for geometric mean of the endogenous control genes, and for a calibrator sample, with the QPCR software^46^. The normalised Cq or log_2_Cq values were used to test expression differences between the cases and controls and, additionally, between the preterm and term placentae utilising non-parametric permutation test (ΔΔCq used) and two-way ANOVA (log_2_ΔΔCq used). Statistical analyses were conducted with the QPCR software and IBM SPSS Statistics 21 software (IBM Corp).

## Additional Information

**How to cite this article**: Kaartokallio, T. *et al.* Gene expression profiling of pre-eclamptic placentae by RNA sequencing. *Sci. Rep.* **5**, 14107; doi: 10.1038/srep14107 (2015).

## Supplementary Material

Supplementary Information

Supplementary Table S3

Supplementary Table S4

Supplementary Table S5

Supplementary Table S7

## Figures and Tables

**Table 1:** Clinical characteristics of the study population.

Maternal or perinatal characteristic	RNA-seq			qPCR validation		
	Controls (n = 9)	Cases (n = 9)	p	Controls (n = 19)	Cases (n = 20)	p
Age (years)	32.3 ± 3.6	34.0 ± 5.5	0.46	30.8 ± 5.2	33.2 ± 5.9	0.20
BMI (kg/m^2^)	21.5 (20.2/24.3)	22.6 (21.7/25.1)	0.26	23.0 (20.3/27.7)	23.3 (21.4/25.6)	0.75
Before pregnancy smokers	3 (33.3%)	1^a^ (12.5%)	0.58	5^a^ (27.8%)	2 (10.0%)	0.22
During pregnancy smokers	0	0^a^	−	1^a^ (5.6%)	0	0.47
Parity						
-Primipara	1 (11.1%)	6 (66.7%)		7 (36.8%)	15 (75.0%)	
-Multipara	8 (88.9%)	3 (33.3%)	0.050	12 (63.2%)	5 (25.0%)	0.016
Systolic blood pressure (mmHg)	121.0 (119.5/126.0)	168.0 (154.5/179.5)	<0.001	121.0 (117.0/126.0)	168.5 (159.3/174.8)	<0.001
Diastolic blood pressure (mmHg)	80.0 (76.5/83.5)	111.0 (101.0/117.5)	<0.001	80.0 (77.0/83.0)	110.0 (105.0/118.3)	<0.001
Proteinuria (g/24 h)	−	3.43 (2.19/5.65)	−	−	4.19 (1.40/8.27)	−
Gestational diabetes	0	0	−	2 (10.5%)	2 (10.0%)	1.00
Gestational age at birth (weeks)	39.1 (39.1/39.4)	36.0 (33.4/38.3)	0.006	39.0 (35.1/39.3)	36.4 (33.0/39.2)	0.18
Relative birth weight	0.62 (−0.63/1.83)	−1.97 (−2.38/−0.58)	0.019	−0.33 (−1.1/0.62)	−1.43 (−1.99/−0.56)	0.003
Method of delivery						
-Vaginal	0	0		7 (36.8%)	7 (35.0%)	
-C-section	9 (100%)	9 (100%)	−	12 (63.2%)	13 (65.0%)	0.91
Contractions	0	0	−	7 (36.8%)	8 (40.0%)	0.84
Sex of child						
-Boy	5 (55.6%)	4 (44.4%)		8 (42.1%)	11 (55.0%)	
-Girl	4 (44.4%)	5 (55.6%)	1.0	11 (57.9%)	9 (45.9%)	0.42
Delay from detachment of placenta to sampling (min)	23.0 (18.5/26.0)	43.0 (25.5/57.5)	0.008	23.0 (18.0/29.0)	31.5 (24.3/44.5)	0.008

Continuous variables: data are presented as mean ± standard deviation for the normally distributed data (maternal age) and as median (25th/75th percentile) for the data that is non-normally distributed in at least one of the sample sets (BMI, blood pressure, gestational age at birth, relative birth weight and delay from detachment of placenta to 9-site sampling). Variables were compared using an independent samples t-test for parametric data and an independent samples Mann-Whitney U test for non-parametric data. Discontinuous variables: data are presented as frequencies (%). Variables were compared using chi square test or Fisher’s exact test for the variables with less than five observations in at least one cell. BMI: body mass index; C-section: Caesarean section.

^a^information missing for one study subject.

**Table 2:** The genes with altered expression in pre-eclamptic placentae in the RNA-seq.

Gene symbol	Function	Protein localisation	Log_2_ FCAll pools	Log_2_ FCw/o PE1	Log_2_ FCw/o PE3	Up/Down
*ACTA2*	Smooth muscle cell contractility	Cytoplasm		−1.31		↓
*ARMS2*	Unknown	Cytoplasm	2.36	2.26	1.98	↑
*ARNT2*	Response to hypoxia	Nucleus		1.50		↑
*BHLHE40*	Cell differentiation, growth suppression	Nucleus		1.10		↑
*BTNL9*	Regulation of immune response	Membrane		1.82		↑
*CCSAP*	Cilia function, development	Cytoplasm		1.20		↑
*CGB*	Maintenance of pregnancy	Secreted	1.82	1.33	2.21	↑
*CGB1*	Possibly implantation	Secreted	1.30		1.39	↑
*CGB2*	Possibly implantation	Secreted	1.34		1.47	↑
*CGB8*	Maintenance of pregnancy	Secreted	1.22	1.15		↑
*COL17A1*	Cell adhesion, negative regulation of cell motility	Membrane, secreted		1.10		↑
*CORO2A*	Inflammatory response	Cytoplasm		1.86		↑
*CXCL9*	T-cell chemoattractant	Secreted	−3.10	−3.04	−2.93	↓
*CYP1A1*	PAH metabolism	Cytoplasm			−5.12	↓
*DEFA1*	Host defence	Cytoplasm, secreted	−2.69	−3.18		↓
*DEFA1B*	Host defence	Cytoplasm, secreted	−2.68	−3.17		↓
*DIO2*	Activation of thyroid hormone	Cytoplasm		1.59		↑
*FCGR3A*	Antibody-dependent responses, natural killer cell cytotoxicity	Membrane, secreted	−1.31	−1.26	−1.28	↓
*FER1L4*	Long noncoding RNA, gene expression regulation	No protein product			0.96	↑
*FSTL3*	Regulation of growth factor activity during development, metabolic homeostasis	Secreted	1.35	1.50	1.33	↑
*HBB*	Component of adult hemoglobin	Cytoplasm	−1.20	−1.62		↓
*HBG1*	Component of fetal hemoglobin	Cytoplasm			−1.12	↓
*HK2*	Glucose metabolism	Cytoplasm	1.72	2.09		↑
*HLA-DRA*	Alpha subunit of HLA-DR, presenting antigens to T-cells	Membrane	−1.26	−1.30	−1.17	↓
*HPSE*	Remodelling of extra-cellular matrix to permit cell movement	Secreted		1.08		↑
*HTRA4*	Degradation of misfolded proteins	Secreted	1.32	1.63	1.03	↑
*IGFBP1*	Binds to insulin-like growth factors, possibly restricts fetal growth	Secreted			1.29	↑
*KRT6A*	Epithelial structures, wound healing	Cytoplasm	−5.34	−5.41	−4.92	↓
*LEP*	Fetal growth, reproduction, angiogenesis, suppression of appetite, regulation of immune response	Secreted	2.26	2.67		↑
*LGALS14*	Trophoblast differentiation	Nucleus	−0.98	−1.05		↓
*LOC100129345*	Unknown	No protein product	−1.09		−1.35	↓
*LYN*	Immune response, response to growth factors, and cell proliferation, survival, differentiation, and migration	Cytoplasm		1.05		↑
*LYPD5*	Unknown	Membrane		−1.28		↓
*LYZ*	Antibacterial activity	Secreted	−1.22		−1.22	↓
*MAGEA4*	Promotion of cell growth	Cytoplasm			−1.71	↓
*MYO7B*	Transport	Cytoplasm	1.71	1.82		↑
*NCMAP*	Myelination	Membrane			−0.99	↓
*NDRG1*	Stress and hormone response, cell growth, differentiation, apoptosis	Nucleus		1.08		↑
*NRIP1*	Modulation of transcriptional activity	Nucleus		1.31		↑
*PLIN2*	Lipid accumulation, inhibition of cellular glucose uptake	Cytoplasm		1.28		↑
*PSG10P*	Unknown	No protein product			−1.15	↓
*PVRL4*	Cell adhesion	Membrane, secreted		1.24		↑
*RPS17*	A component of the ribosomal 40S subunit	Cytoplasm	−1.40	−1.37	−1.34	↓
*S100A8*	Multiple functions in immune response, antioxidant function	Cytoplasm, secreted		−1.78		↓
*SASH1*	Inhibition of invasion, growth and proliferation, proinflammatory	Intracellular	1.23	1.45		↑
*SBSPON*	Unknown	Secreted		−2.28		↓
*SERPINB9*	GranzymeB inhibition	Cytoplasm	0.98	1.28		↑
*SH3BP5*	Neuroprotection, reduction of oxidative stress	Cytoplasm		1.11		↑
*SIGLEC6*	Binds leptin, affects proliferation, invasion and apoptosis	Membrane, secreted		1.33		↑
*TET3*	DNA methylation process, epigenetic chromatin reprogramming	Nucleus	1.30	1.61		↑
*THY1*	T-cell cell surface glycoprotein	Membrane		−1.30		↓
*TPBG*	Promigratory	Membrane		1.05		↑
*ZNF175*	Suppression of viral replication	Nucleus		1.30		↑

Log_2_ fold change (FC) is shown for the subanalyses where a trancript had a q value <0.05. PE = pre-eclampsia.

**Table 3:** Pathway analysis results from Gene Set Enrichment Analysis.

Pathway	ES (+/−)	FDR q value		
		All pools	w/o PE1	w/o PE3
Allograft rejection	−	<0.001	<0.001	<0.001
Interferon gamma response	−	<0.001	<0.001	<0.001
Inflammatory response	−	<0.001	<0.001	0.002
Kras signalling up	−	<0.001	<0.001	0.002
Complement	−	0.001	0.011	0.002
IL6 JAK STAT3 signaling	−	0.001	0.001	0.009
Interferon alpha response	−	0.002	<0.001	0.018
Epithelial mesenchymal transition	−	0.009	0.001	
Oxidative phosphorylation	−	0.022		0.001
Xenobiotic metabolism	−	0.023		
Coagulation	−	0.026	0.038	
Angiogenesis	−	0.027	0.004	
Bile acid metabolism	−	0.035		
TNFa signalling via NFKb	−	0.034		
Reactive oxygen species pathway	−	0.038		
IL2 STAT5 signaling	−	0.048		0.034
Protein secretion	−			0.009

The hallmark gene sets were utilised in the analyses. ES = Enrichment score; a negative ES indicates gene set enrichment at the bottom of the ranked list i.e. the genes that were down-regulated in pre-eclampsia. FDR q value = a false discovery rate corrected p value, q values < 0.05 are shown in the table.

**Table 4:** Quantitative PCR validation of 11 genes with altered expression in pre-eclamptic placentae in the RNA-seq.

Gene	Control vs. pre-eclampsia			Term vs. preterm			Up/Dowm*
	log_2_FC	Permutation test p value^1^	ANOVA p value^2^	log_2_FC	Permutation test p value^1^	ANOVA p value^2^	
Expression affected by pre-eclampsia status							
*LEP*	2.81	**<0.001**	**<0.001**	1.00	0.255	0.156	↑
*HTRA4*	1.96	**<0.001**	**<0.001**	1.80	**0.001**^3^	**0.017**^3^	↑
*SASH1*	1.01	**<0.001**	**0.003**	0.65	0.110	0.077	↑
*CCSAP*	0.64	**<0.001**	**0.005**	0.06	0.781	0.672	↑
*PLIN2*	0.54	**0.037**	**0.036**	0.36	0.174	0.131	↑
Expression affected by gestational age							
*DEFA1/DEFA1B*	−1.28	**0.028**	0.098	−1.49	**0.010**	**0.005**	↓
*CGB*	0.27	0.446	0.478	1.07	**<0.001**	**0.003**	↑
*LYZ*	0.10	0.756	0.541	−0.81	**0.003**	**0.005**	↓
*FCGR3A*	−0.13	0.513	0.659	−0.45	**0.020**	**0.029**	↓
*LGALS14*	−0.34	0.122	0.082	−0.45	**0.043**	0.067	↓
Expression affected by pre-eclampsia status and gestational age							
*TPBG*	0.89	**<0.001**	**0.004**	0.66	**0.011**	**0.011**	↑ PE ↓ preterm

FC: fold change; gw: gestational week; PE: pre-eclampsia. The validation sample consists of 20 pre-eclamptic and 19 non-pre-eclamptic women, including 8 pre-eclamptic and 9 non-pre-eclamptic women from the RNA-seq.

^1^Non-parametric permutation test conducted to compare control and pre-eclampsia, and term (gw ≥ 37 + 0) and preterm (gw < 37 + 0) separately. Cq values normalised for geometric mean of reference genes and a calibrator (ΔΔCq) were used in the analysis.

^2^Two-way ANOVA with categorical variables pre-eclampsia status (control/pre-eclampsia) and gestational age (<37 + 0/ ≥ 37 + 0). Log_2_ ΔΔCq values were utilised in the analysis.

^3^Gestational age affects expression level only in the pre-eclamptic group.

^4^in pre-eclamptic placenta compared to control placenta or in preterm placenta (gw < 37 + 0) compared to term placenta (gw ≥ 37 + 0).
