# Corrigendum: Gene expression profiling of pre-eclamptic placentae by RNA sequencing

**DOI:** 10.1038/srep17245

**Published:** 2016-01-07

**Authors:** Tea Kaartokallio, Alejandra Cervera, Anjuska Kyllönen, Krista Laivuori, Juha Kere, Hannele Laivuori, The FINNPEC Core Investigator Group, Hannele Laivuori, Seppo Heinonen, Eero Kajantie, Juha Kere, Katja Kivinen, Anneli Pouta

Scientific Reports 5: Article number: 1410710.1038/srep14107; published online: 09212015; updated: 01072016.

The Acknowledgements section in this Article is incomplete.

“We are indebted to all the FINNPEC study participants. We appreciate the contribution of the following members of the FINNPEC Study Group: Eeva Ekholm (Turku University Central Hospital, Turku, Finland), Kaarin Mäkikallio-Anttila, (Oulu University Hospital, Oulu, Finland), Reija Hietala, Susanna Sainio and Terhi Saisto (Helsinki University Hospital, Helsinki, Finland), Tia Aalto-Viljakainen, Sanna Heino and Anna Inkeri Lokki (University of Helsinki, Helsinki, Finland), and Leena Georgiadis (Kuopio University Hospital, Kuopio, Finland). We also thank Sampsa Hautaniemi (University of Helsinki, Helsinki, Finland) for his collaboration. The expert technical assistance of Katariina Hirvonen, Elina Huovari, Eija Kortelainen, Satu Leminen, Aija Lähdesmäki, Susanna Mehtälä, and Christina Salmen is gratefully acknowledged. We would also like to acknowledge support from Science for Life Laboratory, the Swedish national infrastructure SNISS, and Uppmax for providing assistance in massively parallel sequencing and computational infrastructure.”

should read:

“We are indebted to all the FINNPEC study participants. We appreciate the contribution of the following members of the FINNPEC Study Group: Eeva Ekholm (Turku University Central Hospital, Turku, Finland), Kaarin Mäkikallio-Anttila, (Oulu University Hospital, Oulu, Finland), Reija Hietala, Susanna Sainio and Terhi Saisto (Helsinki University Hospital, Helsinki, Finland), Tia Aalto-Viljakainen, Sanna Heino and Anna Inkeri Lokki (University of Helsinki, Helsinki, Finland), and Leena Georgiadis (Kuopio University Hospital, Kuopio, Finland). We also thank Sampsa Hautaniemi (University of Helsinki, Helsinki, Finland) for his collaboration. The expert technical assistance of Katariina Hirvonen, Elina Huovari, Eija Kortelainen, Satu Leminen, Aija Lähdesmäki, Susanna Mehtälä, and Christina Salmen is gratefully acknowledged. We would also like to acknowledge support from Science for Life Laboratory, the Swedish national infrastructure SNISS, and Uppmax for providing assistance in massively parallel sequencing and computational infrastructure.

This work was supported by Academy of Finland; Jane and Aatos Erkko Foundation; Päivikki and Sakari Sohlberg Foundation; Research Funds of the University of Helsinki; Government Special state subsidy for Health Sciences (EVO funding) at Helsinki and Uusimaa Hospital District; Novo Nordisk Foundation; Finnish Foundation for Pediatric Research; Emil Aaltonen Foundation; Sigrid Jusélius Foundation; Biocentrum Helsinki to AC; Doctoral Programme in Biomedicine (DPBM) to AC and TK; Doctoral Programme in Clinical Research (KLTO) to TK, Research Foundation of the University of Helsinki to TK and Biomedicum Helsinki Foundation to TK.”

