# Supplementary material for: Gene expression profiling of pre-eclamptic placentae by RNA sequencing
Source: Sci Rep. 2015 Sep 21;5:14107. doi: 10.1038/srep14107 (PMC4585671; doi:10.1038/srep14107)

Gene expression profiling of pre-eclamptic placentae by RNA sequencing

Tea Kaartokallio[1]*, Alejandra Cervera[2], Anjuska Kyllönen[1], Krista Laivuori[1,3,4], Juha Kere[3,4,5], Hannele Laivuori[1,6,7], The FINNPEC Core Investigator Group

1 Medical Genetics, University of Helsinki and Helsinki University Hospital, Helsinki, FI-00014, Finland

2 Research Programs Unit, Genome-Scale Biology and Institute of Biomedicine, University of Helsinki, Helsinki, FI-00014, Finland

3 Molecular Neurology Research Program, University of Helsinki, Helsinki, FI-00014, Finland

4 Folkhälsan Institute of Genetics, Helsinki, FI-000290, Finland

5 Department of Biosciences and Nutrition, Center for Innovative Medicine, and Science for Life Laboratory, Karolinska Institutet, Stockholm, Sweden

6 Obstetrics and Gynaecology, University of Helsinki and Helsinki University Hospital, University of Helsinki, Helsinki, FI-00014, Finland

7 Institute for Molecular Medicine Finland, University of Helsinki, Helsinki, FI-00014, Finland

*tea.kaartokallio@helsinki.fi

<u>Members of the FINNPEC Core Investigator Group:</u>

Hannele Laivuori, Seppo Heinonen, Eero Kajantie, Juha Kere, Katja Kivinen, and Anneli Pouta

SUPPLEMENTARY MATERIAL

Supplementary Table S1. Quality metrics for the RNA-seq data

Pool (F/R)	Read count before filtering	% of reads filtered	Read count*	Read length*	GC%*	Duplicate reads (%)*
PE 1_F	31211527	1.6	30701210	25-101	49	65.9
PE 1_R	31211527	1.6	30701210	25-101	49	65.3
PE 2_F	29927649	1.9	29358515	25-101	50	67.0
PE 2_R	29927649	1.9	29358515	25-90	50	64.6
PE 3_F	35701818	1.6	35129518	25-101	48	73.3
PE 3_R	35701818	1.6	35129518	25-90	48	71.1
Ctrl 1_F	32761025	2.0	32100295	25-101	49	69.2
Ctrl 1_R	32761025	2.0	32100295	25-94	49	66.9
Ctrl 2_F	30276310	2.0	29676081	25-101	49	69.9
Ctrl 2_R	30276310	2.0	29676081	25-90	49	67.7
Ctrl 3_F	39860949	2.0	39060015	25-101	48	73.6
Ctrl 3_R	39860949	2.0	39060015	25-90	48	71.4

*After read trimming and filtering
PE=pre-eclampsia, Ctrl=control, F=forward, R=reverse

Supplementary Table S2. Quality metrics for the Tophat alignment

Pool	Mapped reads	Mapped with mate	Mapped without mate	Mate mapped to different Chr*	Unique alignments (%)
PE1	59,423,667	55,851,614 (94.0%)	3,572,053 (6.0%)	629,972 (1.1%)	92.1
PE2	59,182,860	55,509,582 (93.8%)	3,673,278 (6.2%)	680,992 (1.2%)	90.1
PE3	71,275,870	67,655,152 (94.9%)	3,620,718 (5.1%)	767,588 (1.1%)	91.3
Ctrl1	65,262,608	61,488,022 (94.2%)	3,774,586 (5.8%)	721,282 (1.2%)	89.9
Ctrl2	60,435,844	56,941,186 (94.2%)	3,494,658 (5.8%)	622,024 (1.1%)	89.8
Ctrl3	78,035,758	73,779,502 (94.5%)	4,256,256 (5.5%)	876,940 (1.2%)	92.1

*count (percentage) of the reads that mapped with mate
PE=pre-eclampsia, Ctrl=control, Chr=chromosome

Supplementary Table S6. Diseases and functions annotations from functional IPA analysis.

Category	Examples of disease and function annotations within each category	Number (%) of molecules in each category		
		All pools	W/o PE1	W/o PE3
Hematological system development and function	Activation of blood cells, binding of leucocytes, chemotaxis of monocytes, proliferation of immune cells, activation of antigen presenting cells, T cell migration, infiltration of leucocytes, differentiation of blood cells	84 (32.3)	151 (33.0)	67 (27.0)
Immune cell trafficking	Activation of leucocytes, chemotaxis of leucocytes, cell movement of phagocytes, recruitment of granulocytes, lymphocyte migration, NK cell migration	62 (23.8)	109 (23.8)	52 (21.0)
Cellular movement	migration of cells, chemotaxis of cells, cell movement of leucocytes, cell movement of myeloid cells, cell movement of phagocytes, recruitment of granulocytes, migration of cancer cells	70 (26.9)	136 (29.7)	63 (25.4)
Cell-to-cell signaling and interaction	activation of cells, stimulation of cells, binding of blood cells, attraction of cells, intercellular communication, signal transduction, adhesion of immune cells	96 (36.9)	131 (28.6)	73 (29.4)
Inflammatory response	Activation of phagocytes, chemotaxis of mononuclear leucocytes, inflammation of organ, chemotaxis of neutrophils, cell movement of macrophages, inflammation of endothelial cells	83 (31.9)	148 (32.3)	67 (27.0)
Cell death and survival	Killing of cells, cytolysis, cell death of immune cells, necrosis, apoptosis, cell survival, hemolysis	90 (34.6)	157 (34.3)	75 (30.2)
Immunological disease	Systemic autoimmune syndrome, systemic lupus erythematosus, insulin-dependent diabetes mellitus, allergy, Sjögren's syndrome	79 (30.4)	126 (27.5)	66 (26.6)
Cancer	Metastatic carcinoma, metastatic endocrine gland tumor, advanced malignant tumor, ovarian neoplasia, growth of tumor, metastasis	177 (68.1)	318 (69.4)	72 (29.0)
Connective tissue disorders	arthritis, rheumatoid arthritis	61 (23.5)	86 (18.8)	48 (19.4)
Inflammatory disease	chronic inflammatory disorder, dermatitis, inflammatory bowel disease, asthma	80 (30.8)	132 (28.8)	64 (25.8)
Cellular growth and proliferation	Stimulation of cells, proliferation of immune cells, proliferation of cells	91 (35.0)	175 (38.2)	72 (29.0)
Tissue development	Adhesion of blood cells, adhesion of epithelial cells, aggregation of cells, endothelial cell development, growth of epithelial tissue, detachment of epithelial cells, detachment of endothelial cells inflammation of endothelial cells	66 (25.4)	120 (26.2)	46 (18.5)
Cell signaling	mobilization of Ca2+, regulation of hormone, release of nitric oxide	35 (13.5)	68 (14.8)	34 (13.7)
Free radical scavenging	synthesis of reactive oxygen species, release of reactive oxygen species	28 (10.8)	48 (10.5)	20 (8.1)
Molecular transport	flux of inorganic cation, flux of Ca2+, quantity of Ca2+, secretion of molecule	62 (23.8)	104 (22.7)	64 (25.8)
Cardiovascular	vascular disease, disorder of artery, occlusion of	34	80	29

disease	artery, atherogenesis, hemolytic uremic syndrome, atherosclerosis, vascular lesion, hypertension	(13.1)	(17.5)	(11.7)
Carbohydrate metabolism	neutralization of polysaccharide, hydrolysis of polysaccharide, synthesis of polysaccharide, hydrolysis of proteoglycan, quantity of heparin	15 (5.8)	8 (1.7)	26 (10.5)
Cardiovascular system development and function	vascularization, development of blood vessel, angiogenesis, formation of endothelial tube, chemotaxis of endothelial cell lines, development of vascular system, adhesion of endothelial cells, migration of endothelial cells, proliferation of endothelial cells	36 (13.8)	68 (14.8)	31 (12.5)

The table shows the diseases and functions categories that had the largest number of statistically significant annotations in all the three subanalysis (in italics), and additional statistically significant categories selected. For each category, a few examples of annotations are shown. Each annotation might belong to several categories.

Supplementary Table S8. Quantitative PCR validation of 11 genes with altered expression in pre-eclamptic placentae in the RNA-seq. Diabetics have been excluded in this analysis.

Gene	Control vs. pre-eclampsia			Term vs. preterm			Up/Down *
	log_2FC	Permutation test p value[1]	ANOVA p value[2]	log_2FC	Permutation test p value[1]	ANOVA p value[2]	
**Expression affected by pre-eclampsia status**							
LEP	2.90	**<0.001**	**0.001**	1.25	0.173	0.117	↑
HTRA4	1.86	**0.003**	**0.002**	1.99	**<0.001**[3]	**0.005**[3]	↑
SASH1	1.07	**<0.001**	**0.007**	0.73	0.085	0.121	↑
CCSAP	0.57	**0.006**	**0.035**	0.15	0.490	0.467	↑
**Expression affected by gestational age**							
DEFA1/DEFA1B	-1.21	0.051	0.361	-1.71	**0.007**	**0.001**	↓
CGB	0.33	0.399	0.635	1.11	**<0.001**	**0.003**	↑
LYZ	0.15	0.640	0.291	-0.86	**0.003**	**0.004**	↓
FCGR3A	-0.10	0.623	0.902	-0.47	**0.024**	**0.033**	↓
LGALS14	-0.48	**0.048**	0.065	-0.57	**0.019**	**0.042**	↓
PLIN2	0.47	0.094	0.148	0.55	**0.040**	**0.044**	↑
**Expression affected by pre-eclampsia status and gestational age**							
TPBG	0.91	**<0.001**	**0.008**	0.73	**0.008**	**0.016**	↑ PE<br>↓ preterm

After excluding diabetic patients from the analysis, the validation sample consists of 18 pre-eclamptic and 17 non-pre-eclamptic women, including 8 pre-eclamptic and 9 non-pre-eclamptic women from the RNA-seq.

*in pre-eclamptic placenta compared to control placenta or in preterm placenta (gw<37+0) compared to term placenta (gw ≥ 37+0)

[1] Non-parametric permutation test conducted to compare control and pre-eclampsia, and term (gw ≥ 37+0) and preterm (gw<37+0) separately. Cq values normalised for geometric mean of reference genes and a calibrator (ΔΔCq) were used in the analysis.

[2] Two-way ANOVA with categorical variables pre-eclampsia status (control/pre-eclampsia) and gestational age (<37+0/≥ 37+0). Log_2 ΔΔCq values were utilised in the analysis.

[3] Gestational age affects expression level only in the pre-eclamptic group

FC: fold change; gw: gestational week; PE: pre-eclampsia

Supplementary Table S9. Taqman assays and cDNA dilutions for target genes

Gene	Taqman assay	cDNA dilution
FCGR3A	Hs02388314_m1	1:2
CCSAP	Hs00299198_m1	1:5
HTRA4	Hs01059836_m1	1:5
LEP	Hs00174877_m1	1:5
LYZ	Hs00426232_m1	1:5
PLIN2	Hs00605340_m1	1:5
SASH1	Hs01083311_m1	1:5
TPBG	Hs00272649_s1	1:5
CGB	Hs00361224_gH	1:10
DEFA1/DEFA1B/DEFA3	Hs00414018_m1	1:10
LGALS14	Hs00944203_g1	1:10
*TBP**	Hs00427621_m1	1:2 / 1:5 / 1:10
*YWHAZ**	Hs03044281_g1	1:2 / 1:5 / 1:10

*Endogenous control gene

Supplementary Figure S1. The overlap in the genes with a $\log_2$ fold change ≥ 1 or ≤ -1 between the subanalyses. PE= pre-eclampsia.

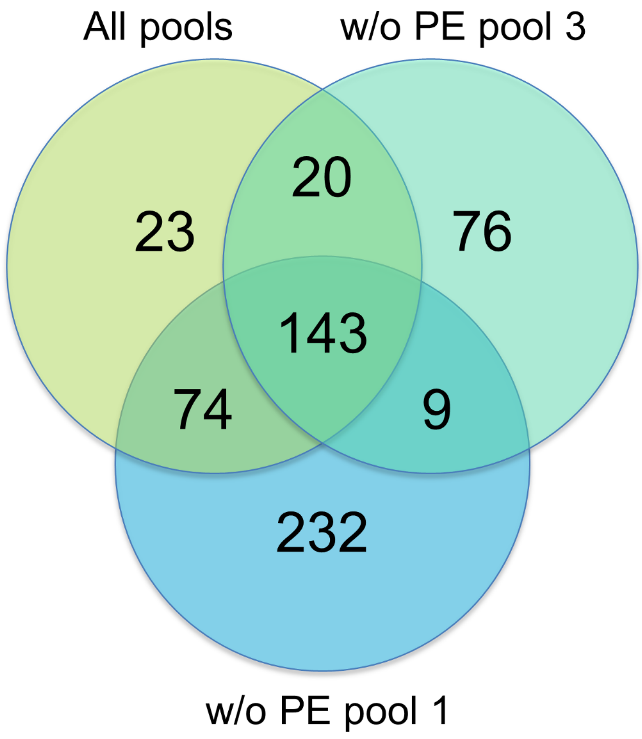

Supplement: Supplementary Information [file srep14107-s1.pdf]
